# Procalcitonin Predicts Real-Time PCR Results in Blood Samples from Patients with Suspected Sepsis

**DOI:** 10.1371/journal.pone.0053279

**Published:** 2012-12-27

**Authors:** Antonella Mencacci, Christian Leli, Angela Cardaccia, Marta Meucci, Amedeo Moretti, Francesco D'Alò, Senia Farinelli, Rita Pagliochini, Mariella Barcaccia, Francesco Bistoni

**Affiliations:** Microbiology Section, Department of Experimental Medicine and Biochemical Sciences, University of Perugia, Perugia, Italy; Charité, Campus Benjamin Franklin, Germany

## Abstract

**Background:**

Early diagnosis and rapid bacterial identification are of primary importance for outcome of septic patients. SeptiFast® (SF) real-time PCR assay is of potential utility in the etiological diagnosis of sepsis, but it cannot replace blood culture (BC) for routine use in clinical laboratory. Procalcitonin (PCT) is a marker of sepsis and can predict bacteremia in septic patients. The aim of the present study was to investigate whether PCT serum levels could predict SF results, and could help screening febrile patients in which a SF assay can improve the etiological diagnosis of sepsis.

**Methods:**

From 1009 febrile patients with suspected sepsis, 1009 samples for BC, SF real-time PCR, and PCT determination were obtained simultaneously, and results were compared and statistically analysed. Receiver operating characteristic (ROC) curves were generated to determine the area under the curve and to identify which cut-off of PCT value produced the best sensitivity to detect SF results.

**Results:**

Mean PCT values of sera drawn simultaneously with samples SF positive (35.42±61.03 ng/ml) or BC positive (23.14±51.56 ng/ml) for a pathogen were statistically higher than those drawn simultaneously with SF negative (0.84±1.67 ng/ml) or BC negative (2.79±16.64 ng/ml) samples (p<0.0001). For SF, ROC analysis showed an area under the curve of 0.927 (95% confidence interval: 0.899–0.955, p<0.0001). The PCT cut-off value of 0.37 ng/ml showed a negative predictive value of 99%, reducing the number of SF assays of 53.9%, still identifying the 96.4% of the pathogens.

**Conclusion:**

PCT can be used in febrile patients with suspected sepsis to predict SF positive or negative results. A cut-off value of 0.37 ng/ml can be considered for optimal sensitivity, so that, in the routine laboratory activity, SF assay should not be used for diagnosis of sepsis in an unselected patient population with a PCT value <0.37 ng/ml.

## Introduction

Sepsis is a major threat to life [1]. Early diagnosis and rapid bacterial identification are of primary importance for a correct clinical management and initiation of effective antimicrobial therapy [2]–[4].

Blood culture (BC) is considered the gold standard for detection of pathogens from patients with sepsis, but it remains insufficiently time critical and cannot assist with early therapeutic decisions [5].

Molecular assays are of potential utility in improving the diagnosis of sepsis. Recently, SeptiFast® (SF), a multi-pathogen probe-based real-time PCR system targeting DNA sequences of bacteria and fungi present in blood samples [6], has been intensively investigated in clinical studies and is considered a valuable complementary tool in the management of patients with suspected sepsis [7]–[9]. However, it provides information not entirely interchangeable with BC and it is not superior to BC for pathogen identification, in an unselected patient population with suspected sepsis [10]. In addition, this molecular assay is expensive, time-consuming, and requires special equipment and technical expertise for DNA extraction. These limitations make it not suitable as replacement of BC for routine use in clinical laboratory, but useful in selected and critically ill patients.

Procalcitonin (PCT), a 116 amino acid peptide precursor of calcitonin [11], increases during various forms of inflammation and infections [12] and correlates with the extent and severity of bacterial invasion [13]–[15]. Among early diagnostic markers of sepsis, PCT seems to have a good diagnostic accuracy in predicting bacteremia [16], [17].

The aim of the present study was to investigate whether PCT serum levels could predict SF results, and could help to select febrile patients needing a SF assay to be performed when a rapid etiologic diagnosis of sepsis is required.

## Methods

### Ethics Statement

All data were analyzed anonymously, and approval for the study was obtained from the Comitato Etico Aziende Sanitarie Umbria.

### Patients and samples

This study was conducted using routine laboratory data collected from the Clinical Microbiology Unit of the General Hospital of Perugia, Italy, from January 2011 to March 2012. Data were from 1009 patients, for which blood culture, SF real-time PCR assay and PCT determination were requested.

Inclusion criteria were: blood samples for BC, SF real-time PCR, and PCT measurement, collected simultaneously from each patient with fever (body temperature ≥37°C) and suspected sepsis, for whom BC and SF were performed for causative pathogen identification. For each patient, only the first samples for BC, SF, and PCT, collected during the same febrile episode were considered. Exclusion criteria were: body temperature <37°C, lack of at least one of the three above samples or samples not drawn simultaneously from the same patient. Samples included in the study were 1009 BC, an equal number of 3-ml K-EDTA-blood samples for SF real-time PCR, and 1009 serum samples for PCT determination.

For each patient included in the study, data on gender, age, ward and time of hospitalization, body temperature and SIRS criteria, antibiotic therapy, and hospital mortality were recorded.

### PCT determination

PCT levels were measured in sera via the automatic analyser VIDAS® B.R.A.H.M.S. PCT assay (bioMérieux, Marcy L'Etoile, France). The lower limit of detection of the assay was 0.05 ng/ml and the functional assay sensitivity was 0.09 ng/ml (VIDAS® B.R.A.H.M.S. PCT package insert; bioMérieux).

### SeptiFast real-time PCR

For SF real-time PCR (Roche Diagnostics GmbH, Mannheim, Germany), a 3-ml K-EDTA-blood sample was collected, and 1.5 ml was processed according to the manufacturer's instructions. Mechanical lysis using the SF Lys kit MGRADE and the MagNA Lyser was performed. Using the SF Prep kit MGRADE, DNA was extracted as described by the manufacturer. Hybridization probes were used. An internal extraction and amplification control, included in the kit, was introduced into each specimen during the first steps of the extraction procedure. This control consists of a mixture of synthetic double-stranded DNA molecules with primer binding sites identical to those of the target sequences but differing in their probe binding sites, thus allowing differentiation of the amplified internal control and the target-specific amplicon. A negative control supplied by the manufacturer was included in each extraction series. Using the LightCycler SF kit MGRADE, real-time PCR was performed in a LightCycler 2.0 instrument (Roche Diagnostics). Three different primer mixes were used to amplify Gram-positive bacteria, Gram-negative bacteria, and fungi. The internal transcribed spacer region was the specific target for the detection of bacterial and fungal pathogens. Reagent controls provided in the kit were used as the positive control of the PCRs. The emitted fluorescence was measured in one of the four different detection channels (610, 640, 670, and 705 nm). Species identification (melting temperature analysis of specimens and controls in each channel) and report generation was obtained using the SF identification software SIS (Roche Diagnostics). The limits of detection were 100 cells/ml for coagulase-negative staphylococci (CoNS) and *Streptococcus* spp. and 30 cells/ml for all other pathogens (LightCycler SF package insert; Roche Diagnostics). The microorganisms identified by SF have been listed elsewhere [6].

### Blood culture

For each sample, an aliquot of 5 to 10 ml whole blood was inoculated into BACTEC® aerobic and anaerobic bottles (Becton Dickinson, Sparks, MD). BACTEC Plus bottles were used for patients under antibiotic therapy and standard bottles for untreated patients. Two sets from two different sites were collected at the same time. The bottles were then incubated in a BACTEC FX (Becton Dickinson) automated blood culture system. All bottles flagged positive were removed from the instrument and an aliquot was taken for Gram stain and culture on solid media for subsequent analysis. Identification of microorganisms was performed with conventional methods and with the Phoenix automatic system (Becton Dickinson).

### Definition of pathogen and sepsis

Microorganisms detected by BC and SF were considered to be pathogens if the results of BC analysis and PCR assay coincided. If SF and BC were positive for different microorganisms, or if a microorganism was only detected by one of the two tests, culture results from other samples, collected from the same patient during the same infectious episode, were evaluated. If these culture tests revealed the presence of the same organism, it was considered a pathogen. If a microorganism was detected without any other culture support, it was considered a pathogen or a contaminant according to the attending physician decision, based on clinical and laboratory signs of sepsis [1] and if the species was generally accepted as a common etiologic agent of the patient's type of infection. In particular, coagulase-negative staphylococci (CoNS), *Corynebacterium* species, and *Propionibacterium acnes*, were considered contaminants when isolated from only one set of BC [18], and in the absence of clinical and/or laboratory data suggesting their pathogenetic role.

Systemic inflammatory response syndrome (SIRS) and sepsis were defined according to the ACCP/SCCM Consensus Conference Committee definition of infection [1].

A bloodstream infection was considered as nosocomial if it occurred ≥48 h after hospital admission [19].

### Statistical analysis

SPSS statistical package, release 13.0 (SPSS Inc, Chicago, IL, USA) was used for all statistical analyses. Values are expressed as mean ± SD. The significance level was 0.05. Mean PCT values were compared across groups using the Mann–Whitney U test for two-group comparisons, and by the Kruskal–Wallis test for multigroup comparisons. The McNemar test was used for testing the differences between paired proportions. Receiver operating characteristic (ROC) curves were generated to determine the area under the curve and to identify which cut-off of PCT value produced the best sensitivity to detect a positive SF or BC result.

## Results

Procalcitonin test, SF real-time PCR, and BC were performed in samples collected simultaneously from 1009 patients from January 2011 to March 2012. Nine hundred and sixteen (90.8%) patients were hospitalized in Medical wards, 75 (7.4%) patients in Surgical wards, and 18 (1.8%) patients in Intensive Care Units (ICUs). Among Medical wards patients, 701 (69.5%) were in Internal Medicine, 116 (11.5%) in Infectious Diseases, 36 (3.6%) in Occupational Medicine, 26 (2.6%) in Neurology, 20 (2%) in Cardiology, 4 (0.4%) in Geriatric Medicine, 4 (0.4%) in Nephrology, 3 (0.3%) in Oncology, 3 (0.3%) in Gastroenterology, 2 (0.2%) in Pulmonology, and 1 (0.1%) in Spinal Unit. Among Surgical wards patients, 33 (3.3%) were in Cardiac Surgery, 16 (1.6%) in Neurosurgery, 15 (1.5%) in General Surgery, 6 (0.6%) in Obstetrics, 2 (0.2%) in Plastic Surgery, 2 (0.2%) in Orthopaedics, and 1 (0.1%) in Vascular Surgery.

The median age of the patients was 69 years (Interquartile Range: 51–80), 55% were males. Mean body temperature was 38.1±0.7°C, 82.3% had body temperature ≥38°C. Antimicrobial therapy had already been started 24 hours prior to sampling in 67.7% of the patients. Six hundred twenty five (61.9%) patients fulfilled SIRS criteria, 314 (31.1%) fulfilled sepsis criteria, and 127 (12.6%) died during hospitalization.

Overall, 136 bloodstream infections were diagnosed by BC and/or SF. Rates of nosocomial bloodstream infections were: 23% in Medical wards, 63% in Surgical wards, and 83% in ICUs. One hundred forty pathogens were identified by BC and/or SF, with an overall concordance of 92.4% between the two methods (Table 1). Blood culture resulted positive in 133 samples, identifying 92 pathogens and 41 contaminants (35 CoNS, 4 *Propionibacterium acnes*, and 2 *Corynebacterium* spp). In one case, the same BC resulted positive for two different isolates: *Escherichia coli* and *Klebsiella pneumoniae*. SeptiFast was positive in 108 samples, detecting 111 pathogens, a number significantly higher (p = 0.04) than those detected by BC. In three cases, the same SF sample was positive for two pathogens: *E. coli* and *K. pneumoniae/oxytoca* in two cases, and *Enterobacter cloacae/aerogenes* and *K. pneumoniae/oxytoca* in one case. In four out of 41 samples corresponding to contaminated BC, SF identified five pathogens: one sample was positive for *E. coli* and *K. pneumoniae/oxytoca*, one for *Enterobacter cloacae/aerogenes*, one for *K. pneumoniae/oxytoca*, and one for *Streptococcus* spp.

**Table 1:** One hundred forty pathogens detected by SeptiFast (SF) and/or blood culture (BC) in 1009 patients.

	No. of pathogens detected		
Pathogen	Only by SF	Only by BC	Both methods
*Staphylococcus aureus*	9	5	15
*Streptococcus pneumoniae*	6	0	3
*Streptococcus spp.* 1	3	2	3
*Enterococcus faecalis*	0	1	2
*Enterococcus faecium*	0	0	1
*Enterobacter cloacae/aerogenes*	3	1	3
*Escherichia coli*	10	9	22
*Serratia marcescens*	0	0	1
*Klebsiella pneumoniae/oxytoca*	11	0	4
*Pseudomonas aeruginosa*	5	1	3
Coagulase-negative staphylococci^2^	0	0	5
*Candida albicans*	0	0	1
*Candida krusei*	1	0	0
Sub-total	48	19	63
*Veillonella parvula*	ND^3^	1	0
*Salmonella* group D	ND	1	0
*Peptostreptococcus spp.*	ND	1	0
*Listeria monocytogenes*	ND	1	0
*Kytococcus sedentarius*	ND	1	0
*Hafnia alvei*	ND	1	0
*Corynebacterium jeikeium*	ND	1	0
*Bacteroides fragilis*	ND	2	0
*Clostridium perfringens*	ND	1	0
Total	48	29	63

1 *Streptococcus* spp. group includes *S. agalactiae, S. pyogenes, S. anginosus*, *S. bovis*, *S. constellatus*, *S. cristatus*, *S. gordonii*, *S. intermedius, S. milleri, S. mitis, S. mutans, S. oralis, S. parasanguinis, S. salivarius, S. sanguinis, S. thermophilus, S. vestibularis, S. viridans*.

2 Coagulase-negative staphylococci group includes *S. epidermidis, S. haemolyticus, S. hominis, S. pasteuri, S. warneri, S. cohnii, S. lugdunensis, S. capitis, S. caprae, S. saprophyticus*, and *S. xylosus*.

3 ND, not included in the SF master list [6].

The mean PCT value was 4.64±22.94 ng/ml in the whole population: 4.61±22.98 ng/ml in Medical wards patients, 1.78±4.98 ng/ml in Surgical wards patients, and 18.36±49.22 ng/ml in ICU patients. A statistically significant difference was found between Medical wards Vs ICUs (p = 0.031) and Surgical wards Vs ICUs (p = 0.016).

Mean PCT values of sera corresponding to positive and negative SF samples were 35.42±61.03 ng/ml and 0.84±1.67 ng/ml, respectively (p<0.0001); similarly, mean PCT values of sera corresponding to BC positive and negative for a pathogen were 23.14±51.56 ng/ml and 2.79±16.64 ng/ml, respectively (p<0.0001). The mean PCT value in sera corresponding to 41 contaminated BC was 2.67±10.44 ng/ml; in the subgroup of sera corresponding to positive SF/contaminated BC samples it was significantly higher than that of the subgroup corresponding to negative SF/contaminated BC samples (18.24±28.19 ng/ml Vs 0.62±0.89 ng/ml, respectively, p<0.0001).

**Figure 1:**
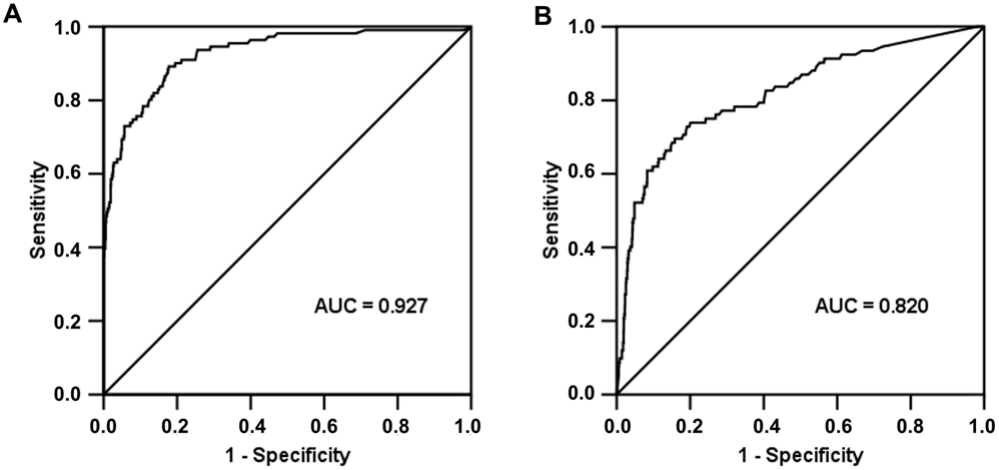
ROC curves for SeptiFast and blood cultures results according to different cut-off of PCT. SeptiFast: Area Under the Curve (AUC) = 0.927; 95% confidence interval (CI): 0.899–0.955, p<0.0001 (panel A). Blood culture: AUC = 0.820; 95% CI: 0.769–0.870, p>0.0001 (panel B).

Receiver Operating Characteristic analysis, performed to assess the diagnostic accuracy of PCT values to predict SF and BC results, showed an area under the curve for SF of 0.927 (95% confidence interval: 0.899–0.955, p<0.0001) and for BC of 0.820 (95% confidence interval: 0.769–0.870, p<0.0001) (Figure 1).

Table 2 shows the sensitivity, specificity, positive and negative predictive values, and positive and negative likelihood ratios of different cut-off values of PCT in predicting SF results. The PCT cut-off value of 0.37 ng/ml seemed to possess the best diagnostic accuracy in predicting SF positive results, with a sensitivity of 96.4%. The cut-off of 0.25 ng/ml was characterized by a slightly higher sensitivity, but lower specificity, positive predictive value, and positive likelihood ratio. Sensitivity of PCT in predicting positive BC results was 82.7% for the cut-off value of 0.37 ng/ml.

**Table 2:** Sensitivity, specificity, positive predictive value, negative predictive value, positive likelihood ratio, negative likelihood ratio of different cut-off values of PCT on SeptiFast results.

Cut-off value of PCT (ng/ml)	Sensitivity	Specificity	PPV	NPV	LR+	LR−
0.05	0.99	0.20	0.13	0.99	1.23	0.05
0.10	0.98	0.36	0.16	0.99	1.53	0.05
0.25	0.98	0.51	0.19	0.99	2.00	0.05
0.37	0.96	0.55	0.21	0.99	2.15	0.05
0.50	0.94	0.67	0.26	0.98	2.84	0.09
1.00	0.90	0.79	0.34	0.98	4.28	0.12
2.00	0.78	0.89	0.47	0.97	7.09	0.24
5.00	0.63	0.97	0.72	0.95	21.0	0.38
10.0	0.46	0.99	0.85	0.94	46	0.54

PPV = positive predictive value.

NPV = negative predictive value.

LR+ = positive likelihood ratio.

LR− = negative likelihood ratio.

Table 3 shows the cost-effectiveness and the number needed to screen for one positive SF or BC test at different PCT cut-off values. Among the 1009 samples, 544 (53.9%) had a PCT <0.37 ng/ml and 465 (46.1%) had a PCT≥0.37 ng/ml. Among the latter, SF detected 107 out the 111 pathogens, missing 2 *Streptococcus* spp. and 2 CoNS pathogens, and BC identified 76 out of 92 pathogens (p<0.0001). Thus, a cut-off value of 0.37 ng/ml would have reduced the total number of SF assays by 53.9%, while still identifying 96.4% of the pathogens. Conversely, at the same or even lower PCT cut-off values, the number of missed pathogens by BC was significantly higher (PCT 0.37 ng/ml: 16 Vs 4, p<0.0001; PCT 0.25 ng/ml: 12 Vs 2, p<0.0001; PCT 0.1 ng/ml: 6 Vs 2, p = 0.009).

**Table 3:** Estimation of costs according to different PCT cut-off values for SeptiFast and blood culture.

		PCT cut-off (ng/ml)						
Variable	No PCT measurement	0.1	0.25	0.37	0.5	1.0	1.5	2.0
**SeptiFast**								
Reduction in sample collection, %	None	30.8	45.6	53.9	60.3	71.2	77.5	81.4
Number of missed pathogens (%)	None	2 (1.8)	2 (1.8)	4 (3.6)	6 (5.4)	10 (9)	19 (17.1)	24 (21.6)
Number needed to screen to detect one pathogen	9.0	6.0	5.0	4.3	3.8	2.9	2.5	2.0
**Blood Culture**								
Reduction in sample collection, %	None	30.8	45.6	53.9	60.3	71.2	77.5	81.4
Number of missed pathogens (%)	None	6 (6.5)	12 (13)	16 (17.4)	20 (21.7)	23 (25.0)	28 (30.4)	31 (33.7)
Number needed to screen to detect one pathogen	11	8.1	6.8	6.1	5.6	4.2	3.5	3.1
**Costs, USD** 1								
Total costs for initial PCT measurement	None	15,640	15,640	15,640	15,640	15,640	15,640	15,640
Total costs for SeptiFast collection	131,170	90,740	71,370	60,450	52,130	37,830	29,510	24,440
SeptiFast total costs per patient	130	105	86	75	67	53	45	40
Total costs for blood culture collection	30,270	20,940	16,470	13,950	12,030	8,730	6,810	5,640
Blood culture total costs per patient	30	36.3	31.8	29.3	27.4	24.2	22.2	21.1

USD = US dollars.

1 For cost calculations, 130 USD for SF, 30 USD per two sets of BC and 15.5 USD per PCT measurement were assumed, based on institutional data of the General Hospital of Perugia, Italy.

## Discussion

Early etiological diagnosis and appropriate therapy are critical to a successful outcome of patients with sepsis. [2]–[4], [20]. Molecular-based technologies are emerging as promising tests as an adjunct to BC in routine clinical laboratory activity to rapidly identify the etiological agents of sepsis [21], [22]. Among these, SF real-time PCR has been the most intensively investigated technique and it is considered a very important tool for the rapid diagnosis of sepsis [8], [23]. However, several limitations of this nucleic-acid based diagnostic technology, such as cost, limited availability, and need of sophisticated equipment, hamper the possibility of using it in the routine laboratory diagnosis in all patients for which a blood culture is required [24]. Nevertheless, in our laboratory practice, we observed that SF test is often requested also in non-septic febrile clinical settings [10], with huge expense, little information, high number of negative results, and consequent low positive predictive value [25]. In this study the majority of the samples were from patients hospitalized in Medical (90.8%) and Surgical (7.4%) wards, while only 1.8% of the samples were from critically ill ICU patients. Moreover, although many patients (61.9%) fulfilled SIRS criteria, only 31.2% of the whole population fulfilled sepsis criteria, with a low positivity rate (10.7%) of SF assay. Thus, a screening assay to predict the probability of positive or negative PCR results, can be of great utility for both microbiologists and clinicians to select patients in which the molecular test can be successfully employed in combination with BC for the etiological diagnosis of sepsis.

Procalcitonin is considered a good biomarker of sepsis that correlates with the extent and severity of infection [14] and a reliable test to rule out or predict bacteremia in patients with acute fever [16], [17] and pneumonia [26]. In line with previous studies [16], [17], [26], also this study shows that PCT is a good biomarker to predict bacteremia, as detected by blood culture. In a study on 79 patients, PCT levels observed in patients with a positive SF assay were higher than those observed in patients with a negative one, but the difference was not statistically significant [27]. Nevertheless, that study evaluated a relatively low number of immunocompromised, mainly onco-haematologic, patients, with a high number of samples negative by BC and positive by SF for fungi, pathogens rarely associated with PCT increase [28]. To our knowledge, this is the first study demonstrating that, in a population composed mainly of patients hospitalized in Internal Medicine wards with fever and suspected sepsis, PCT predicts positive or negative results of SF real-time PCR. This finding seems to be particularly relevant, suggesting that PCT assay, less expensive and more rapid than SF, in the routine clinical and laboratory practice, can be used as a screening test for the SF real-time PCR assay, avoiding a great percentage of unnecessary tests. We found that 96.4% and 98.2% of the SF positive samples corresponded to PCT values≥0.37 ng/ml and ≥0.25 ng/ml, with a 3.6% and 1.8% loss of detection of bacteremia, respectively. Thus, a cut-off value of PCT≥0.37 ng/ml or ≥0.25 ng/ml as a decision rule to perform SF for diagnosis of sepsis, would have resulted in a 53.9% and 45.6% reduction of SF assays, saving 70,720 and 59,800 US dollars, respectively, but still identifying 96.4% and 98.2% of the pathogens detected by SF. These data suggest that the higher sensitivity found for the cut-off of 0.25 ng/ml did not improve the cost-effectiveness of performing SF to additional 84 patients with PCT levels between 0.25 ng/ml and 0.37 ng/ml.

The low specificity, low positive predictive value, and high negative predictive value of PCT found for the cut-off of 0.37 ng/ml can be explained by the low prevalence of positive SF samples (10.7%) and the great number of negative SF samples corresponding to sera with PCT values≥0.37 ng/ml. Indeed, at the cut-off value of 0.37 ng/ml, PCT can be considered a good screening biomarker with high sensitivity, but not a good confirmatory test. Thus, a PCT≥0.37 ng/ml can be used to select samples worth to further the diagnostic process with SF assay. Noteworthy, the cut-off values found for the decision to perform SF assay for the diagnosis of sepsis in this study substantially match those of a PCT-based algorithm for antibiotic therapy decisions described by Schuetz et al. for high-acuity infections patients in ICU settings [29].

Among the 544 samples corresponding to sera with PCT levels <0.37, SF detected four pathogens (two *Streptococcu*s spp. and two CoNS). To go into more depth on this finding, we asked the attending physicians the final diagnosis for the patients these samples come from. Interestingly, all samples were from patients with bacterial endocarditis. This finding is in line with the notion that PCT does not increase significantly in patients with bacterial endocarditis, reaching median values of 0.21 ng/ml [30], and outlines the fact that SF can be successfully used for diagnosis of endocarditis [31].

Procalcitonin may help discriminating blood contamination from bloodstream infection due to CoNS [32]. We found that, in case of BC contamination, PCT levels in sera corresponding to SF positive samples were statistically higher than those corresponding to SF negative ones. Thus, if SF would have been performed only in samples corresponding to PCT≥0.37 ng/ml, all the pathogens (two *K. pneumoniae/oxytoca*, one *E. coli*, one *Enterobacter cloacae/aerogenes spp.*, and one *Streptococcus* spp.) detected in samples corresponding to contaminated BC, would have been identified. On the other hand, SF resulted negative in all cases of BC contaminated by CoNS, *Propionibacterium* spp. and *Corynebacterium* spp., corresponding to PCT <0.37 ng/ml. This is not surprising, given that SF is set with the minimum sensitivity of 100 cells/ml to avoid false positivity due to skin contamination by CoNS or streptococci (Lightcycler SF Package insert, Roche Diagnostics) and *Propionibacterium* spp. and *Corynebacterium* spp. are not included in the SF master list [6].

In the present study, 48 pathogens were detected only by SF and not BC. This discrepancy can be due to the fact that many patients were under antibiotic therapy during sampling, a condition in which SF sensitivity is higher than that of BC [10]. On the other hand, SF failed to detect 29 pathogens identified only by BC. Among them, 10 were not detectable because not included in the SF master list [6], but 19, due mainly to *E. coli* (9 cases) and *S. aureus* (5 cases) pathogens, have to be considered false negative SF results. These may be attributable to a number of factors, including inappropriate sample preparation, low sample volume and microbial load below the detection threshold of the test (30 cells/ml). It is known that bloodstream infections in adults may be characterized by fewer than a single microorganism per 10 ml of blood and that the blood volume drawn greatly affects the diagnostic sensitivity of BC. Particularly, in case of *E. coli* bacteremia, a dramatic increase in pathogen detection was observed with the collection of two sets of BC instead of one [33]. This finding, together with the fact that at 0.37 ng/ml or lower PCT cut-off values, the number of missed pathogens by BC was significantly higher than that of pathogens missed by SF, highlights the notion that BC remains a mainstay for the identification of microorganisms in bloodstream infections and that SF must be considered as an additional tool in selected patients, whenever a quick etiological diagnosis is required for an appropriate and timely clinical management.

In this study, PCT was measured by an enzyme-linked fluorescent immunoassay performed in an automated VIDAS® instrument with a functional assay sensitivity of 0.09 ng/ml. Although this is not the reference automated method, it showed a good correlation and concordance with the established B.R.A.H.M.S PCT KRYPTOR ® assay [34], [35], so that it can be used with the same nominal PCT cut-off ranges in clinical routine [34].

A limitation of this study is the lack of information about the time the blood had been drawn in relation to the fever, as low PCT levels may be present during the first hours of sepsis or as a consequence of effective antimicrobial therapy [14]. However, based on guidelines of our institution, we can assume that the majority of the samples had been collected at the onset of fever. Moreover, the findings that nearly all positive SF and/or BC corresponded to PCT values≥0.37 ng/ml and that the majority of samples were drawn from patients under antibiotic treatment, suggest that, in this study, time of sampling did not affect the results. Nevertheless, this is an important issue that deserves an ad hoc further study.

In conclusion, this study suggests that PCT can be used in an unselected population of patients with fever and suspected sepsis to predict SF positive or negative results. A cut off value of 0.37 ng/ml can be considered for optimal sensitivity. SeptiFast assay should not be performed in febrile patients with suspected sepsis with PCT value <0.37 ng/ml, provided that a bacterial endocarditis is not suspected.
